# Accuracy of self-reported cancer treatment data in young breast cancer survivors

**DOI:** 10.1186/s41687-019-0114-5

**Published:** 2019-05-01

**Authors:** Kelly C. Gast, Elizabeth J. Cathcart-Rake, Aaron Norman, Leah Eshraghi, Nwamaka Obidegwu, Fergus Couch, Celine Vachon, Kathryn J. Ruddy

**Affiliations:** 1 Mayo Clinic, Department of Internal Medicine, 200 First Street SW, Rochester, MN 55095 USA; 2 Mayo Clinic, Department of Oncology, 200 First Street SW, Rochester, MN 55095 USA; 3 Mayo Clinic, Biomedical Statistics and Informatics, 200 First Street SW, Rochester, MN 55095 USA; 4 Dr. Susan Love Research Foundation, 16133 Ventura Boulevard, Suite 1000, Encino, CA 91436 USA; 5 Mayo Clinic, Department of Laboratory Medicine and Pathology, 200 First Street SW, Rochester, MN 55095 USA; 6 Mayo Clinic, Health Sciences Research, 200 First Street SW, Rochester, MN 55095 USA

**Keywords:** Breast cancer, Chemotherapy, Self-reported

## Abstract

**Background:**

Patient-reports of cancer treatments are sometimes used in oncology research and clinically when medical records are unavailable. We aimed to evaluate the accuracy of patient recall in this setting.

**Materials and methods:**

Participants were recruited through an email request from the Dr. Susan Love Research Foundation Army of Women seeking women diagnosed with breast cancer under age 50 and within the past ten years, self-reporting to have been treated with chemotherapy. After informed consent, participants received a web-based survey that inquired about use of and type of chemotherapy and endocrine therapy received. Medical records were reviewed, and discrepancies were defined as patient-report of a different class of drug than documented in the medical record, failing to report a documented class of drug, or responding “don’t know.”

**Results:**

Of 171 eligible participants**,** completed questionnaires and medical records were available for 102 (60%). Median age at diagnosis was 41 years (range 25–49), and median time from diagnosis was 65.5 months (range 7–131). Ninety-two percent had completed college. Receipt of chemotherapy was documented in the medical records of 100% of these women who self-reported a personal history of chemotherapy, and there was also 98% concordance regarding receipt of endocrine therapy (yes vs. no). However, discrepancies were identified in 29% of patients regarding chemotherapy types. Time since diagnosis did not increase the likelihood of discrepancies.

**Conclusion:**

Highly educated young women diagnosed with breast cancer more than five years prior accurately report whether or not they received broad systemic treatment categories. However, self-reports regarding specific drugs should be confirmed by medical record review.

## Introduction

There are approximately 250,000 new cases of breast cancer diagnosed annually in the United States [1], and the American Cancer Society estimates there are more than 3.1 million breast cancer survivors living within the United States [2]. Optimal clinical care and many survivorship research projects rely on understanding the type of treatments these patients receive. However, there are often financial, logistical, and time obstacles to medical record collection and review. Therefore, patient reports are often used in place of medical record reviews in oncologic research [3, 4]. Further, patients who transition care to a new provider may not always bring complete medical records (or time pressure may discourage a provider from completely reviewing a patient’s records), resulting in reliance on patient recall of treatments received.

A few prior studies have assessed the concordance between self-report of breast cancer treatment and medical records [5–10]. These studies have demonstrated high concordance (> 80%) regarding broad categories of treatments received (e.g. surgery, radiation therapy, chemotherapy, or endocrine therapy). For example, one study with a mean time from diagnosis to interview of 19.3 months, found a 98.4% agreement between Iowa SEER Medicare records and patient report of breast cancer treatments in a population of women aged 65+, but that study did not evaluate accuracy regarding specific chemotherapy medications, and younger women were not included [10].

Only three prior studies assessed recall of specific chemotherapy medications received, with variable results [5, 8, 9]. In 895 Australian women diagnosed with breast cancer between 1991 and 1998, agreement regarding specific chemotherapy regimens with a median recall period of 3.2 years ranged from 76 to 93% [5]. Another study of 939 Canadian women diagnosed between 1996 and 1998 with a median recall period of 3.0 years found that agreement rates regarding the specific type of medications received ranged from 90 to 98% for endocrine therapies and from 55 to 89% for chemotherapies [8]. A population-based study of 5042 women diagnosed with breast cancer between 2002 and 2006 in Shanghai, China with a median time from diagnosis of 6.5 months identified that agreement rates between medical records and patient self-report of chemotherapy ranged from 82 to 98% for the ten most commonly prescribed chemotherapy medications [7]. However, breast cancer treatments have become more complex in recent years. For example, trastuzumab +/− pertuzumab are two targeted therapies which are now used in most patients with human epidermal growth factor receptor [Her2]-positive tumors, and new endocrine treatment strategies are available including the combination of an aromatase inhibitor (letrozole, anastrozole, or exemestane) with a gonadotropin-releasing hormone agonist (e.g., goserelin or leuprolide) for women with premenopausal estrogen receptor [ER]-positive disease. None of these prior studies included women diagnosed after 2006. One more recent study that evaluated the accuracy of self-report of fertility-threatening cancer treatments in 101 young cancer survivors of various cancer types including lymphoma, breast cancer, uterine cancer, and ovarian cancer found only a 68% accuracy of reporting exposure to alkylating agents at a median recall period of 2.4 years [11]. The objective of our study was to assess the accuracy of patient self-reports of breast cancer treatments including newer chemotherapy regimens, targeted therapy, and endocrine therapy in a group of young breast cancer survivors.

## Materials and methods

This study was approved by the Mayo Clinic institutional review board. It was conducted in collaboration with the Dr. Susan Love Research Foundation Army of Women, a non-profit organization that connects women and men to breast cancer researchers. Participants were recruited via an email request from the Army of Women in December 2015, seeking women diagnosed with breast cancer under age 50 and within the prior ten years. Those who provided their contact information were mailed a paper consent form and an authorization form to allow study staff to request and review the medical records associated with their cancer diagnosis and treatment. After each participant returned the informed consent, she received a web-based survey link by email. Participants were excluded from the study if they reported a cancer diagnosis other than breast cancer (except they were allowed to participate if the only additional cancer diagnosis they reported was non-melanoma skin cancer, or carcinoma in situ of the cervix because systemic therapies are generally not given for those diagnoses.) Participants were asked about the receipt of chemotherapy, targeted therapy, and endocrine therapy. Those who denied receipt of chemotherapy were excluded.

Participants were then asked to mark the type of chemotherapy, targeted therapy (e.g., trastuzumab), and/or endocrine medication they received with both the generic and brand names listed as options. Common breast cancer drugs and combination regimens were listed. There was also an “other” option with space to specify the drug name and a “don’t know” option. Medical records were requested via fax from the institutions listed on each woman’s authorization form. Medical record abstraction was performed by a single physician using a standardized form.

Medical record data was reviewed for concordance with survey responses. As commonly performed in other research studies, medical record data was considered the gold standard for treatments received [12, 13]. The percentage of patients accurately reporting receipt of common medications and regimens was calculated. Next, all medications reported in the web-based survey were compared to chemotherapy medications documented in the medical record to identify discrepancies. Discrepancies were defined as situations in which the patient reported receipt of a different class of drug than documented in the medical record, the patient responded “don’t know” regarding the specific chemotherapy regimen they received, or the patient failed to report receipt of a medication documented in the medical record. Differences between individual drugs within the same drug class (e.g. docetaxel and paclitaxel) were not considered to be as clinically important, and therefore were not categorized as discrepancies.

## Results

Of the 262 women who completed the web-based survey, 171 were deemed to meet inclusion criteria as specified above, and 102 complete medical records were obtained. Of the 69 participants whose medical records were unable to be analyzed, 40 did not complete the authorization to release protected health information form, 11 medical records were not received from institutions, and 18 medical records did not contain the necessary treatment information. In the eligible 102 participants for whom we had medical records to review, median age at breast cancer diagnosis was 41 years (range 25–49), and median time from diagnosis to survey completion was 65.5 months (range 7–131). All participants were diagnosed between 2005 and 2016. Ninety two percent had completed college, and approximately 48% had completed graduate or professional school (see Table 1). Participants resided in 34 different states with the greatest number of participants from California (11), Virginia (9), New York (7), Massachusetts (5), Wisconsin (5), Missouri (4), Oregon (4), Pennsylvania (4) and Washington (4).

**Table 1:** Patient Characteristics for 102 women diagnosed with breast cancer under 50 years old and reporting chemotherapy

Age at diagnosis	N	%
≤ 30 years	9	9
> 30 years and ≤ 35 years	16	16
> 35 years and ≤ 40 years	25	25
> 40 years and ≤ 45 years	32	31
> 45 years	20	20
Education level		
Graduated from high school or have high school equivalent	2	2
Some college	4	4
Completed vocational/technical training	2	2
Graduated from college	42	41
Some graduate/professional school	3	3
Completed graduate/professional school	49	48
Race (select all that apply)		
American Indian or Alaskan Native	1	1
Asian	4	4
Black or African American	3	3
White	96	94
Estrogen Receptor Status		
Positive	86	84
Negative	16	16
Progesterone Receptor Status		
Positive	74	73
Negative	26	26
Unknown	2	2
Her-2 Neu Status		
Positive	32	32
Negative	67	66
Unknown	2	2
Indeterminate	1	1
Clinical Stage		
0	3	3
1	24	24
2	55	54
3	17	17
Unknown	3	3
Recurrence		
Yes	13	13
No	89	87

Eighty-eight participants (86%) self-reported receipt of anti-estrogen medication, and 34 (33%) self-reported receipt of a targeted therapy. Self-report of chemotherapy treatment was confirmed by medical record review 100% of the time. There was 98% concordance regarding receipt of targeted therapy (yes vs no), and 98% concordance regarding receipt of endocrine therapy (yes vs no). Table 2 presents the proportion of participants who accurately reported receipt of a particular medication. There was 100% concordance between the medical record and web-based survey responses regarding receipt of trastuzumab and pertuzumab. In addition, there was 95% concordance regarding receipt of tamoxifen, but reporting was less accurate for other hormonal medications including letrozole, anastrozole, exemestane, leuprolide, and goserelin (for which accuracy rates were 76%, 62%, 60%, 88%, and 71%, respectively).

**Table 2:** Accuracy of patient-reported receipt of specific medication

Chemotherapy	N	% Correct
Doxorubicin/cyclophosphamide	5	60
AC-T, TAC, or AC-D	57	95
TC	30	77
CMF	1	100
Carboplatin	12	92
Paclitaxel	14	29
Capecitabine	3	67
Abraxane	3	0
Targeted Therapy		
Trastuzumab	33	100
Pertuzumab	9	100
Bevacizumab	1	100
Niratinib	1	0
Palbociclib	1	0
Hormonal Therapy		
Tamoxifen	83	95
Letrozole	21	76
Anastrozole	24	62
Exemestane	10	60
Leuprolide	8	88
Goserelin	7	71

With regard to chemotherapies, women were accurately able to report receipt of the most common regimen, doxorubicin and cyclophosphamide with paclitaxel or docetaxel (AC-T, TAC, or AC-D) with approximately 95% accuracy. Reporting was less accurate (only 77%) for docetaxel and cyclophosphamide (TC). The most common discrepancy was patient reporting receipt of carboplatin when the medical record indicated docetaxel and cyclophosphamide. Table 3 describes the frequency of discrepancies according to patient characteristics including time from diagnosis to survey, age at time of survey, level of education, and stage of breast cancer. Time between diagnosis and survey, age at time of survey, level of education, and stage of breast cancer were not associated with frequency of discrepancies. A total of 6 participants (6%) responded “don’t know” with regard to the type of chemotherapy they received.

**Table 3:** Frequency of discrepancies based on patient characteristics

Characteristic	N	% with a discrepancy in chemotherapy type identified
Time between diagnosis and survey		
≤ 5 years	47	28%
5+ years	55	31%
Age at survey		
≤ 45 years	50	36%
> 45 years	52	23%
Education		
High school grad or equivalent	8	25%
College grad	45	29%
Grad School	49	31%
Stage		
0	3	67%
1	24	33%
2	55	29%
3	17	24%
Unknown	3	0%

## Discussion and conclusions

Patient self-reports of breast cancer treatments are sometimes used in the clinical care of survivors, and commonly used in survivorship research. Our results suggest that this may be particularly useful when the research investigation only requires general information about treatments received, as we found good concordance between medical records and patient reports of broad categories of treatments, consistent with prior studies [5–10]. However, accuracy of self-report of specific medications was imperfect even in those < 5 years from diagnosis, and even in this highly educated population who volunteered to participate in a web-based survey focused on recipients of chemotherapy. This is important for clinicians providing survivorship care to realize as it emphasizes the importance of obtaining medical record confirmation of patient-reported drug data. This also highlights the potential value of survivorship care plans as a mechanism to improve patient recall of treatments received and to enhance communication to future providers to inform clinical care (for example, if an otherwise healthy survivor is experiencing mild intermittent lower extremity edema, cardiac dysfunction will be lower on the differential diagnosis if it is known that a patient was not previously treated with a potentially cardiotoxic drug).

Importantly, our post-diagnosis median recall time (more than five years) was significantly longer than in most other studies. Given that breast cancer survivors are living longer as treatment advances improve cure rates, the demonstration of accurate recall over many years will be important for both clinical care and epidemiological studies focused on long term prognosis, side-effects, and quality of life metrics.

It is possible that discrepancies between patient-reports and medical records may be attributed to recall bias, question design, or missing information from the medical records. A prior study demonstrated lower rates of concordance when open-ended questions were utilized rather than prompts [8], so we provided a prompting list of common breast cancer drugs and combination regimens to aid recall. To the best of our knowledge, this is the first study that analyzed accuracy of self-report of breast cancer treatments utilizing an email request recruitment strategy and a web-based survey. While our study did not demonstrate an association between frequency of discrepancies and education level, our population was overall highly educated, likely at least in part due to our recruitment strategy through Army of Women and use of a web-based survey. Therefore, our results may not be generalizable to less educated individuals. In fact, a prior study performed in low-income women did demonstrate an association between decreased accuracy of self-reports and less education [7].

Other limitations include our relatively small sample size, the use of a single physician for medical record abstraction, and the inability to obtain medical records for all survey respondents. It is possible that discrepancy rates would have been different in those for whom medical records were not provided. We also did not collect medical records for patients who reported that they had not received chemotherapy because the other endpoints of our study (not presented in this manuscript) are related to chemotherapy toxicity, so we are not able to assess rates of underreporting for the yes vs. no chemotherapy variable.

In conclusion, highly-educated young women accurately recall receipt of broad categories of breast cancer treatment including systemic chemotherapy, targeted therapy, and hormonal therapy up to ten years after diagnosis. Therefore, the use of self-report may be an acceptable alternative to medical record abstraction in certain circumstances, but to provide optimal clinical care knowledge of specific medications received, self-report should still be confirmed by medical record review.

## Abbreviations

AC-D: Doxorubicin and cyclophosphamide before or after docetaxel
AC-T: Doxorubicin and cyclophosphamide before or after paclitaxel
CMF: Cyclophosphamide, methotrexate, and 5-fluorouracil
TAC: Doxorubicin and cyclophosphamide concurrent with a taxane
TC: Docetaxel and cyclophosphamide
